# Next-Generation Vaccine Platforms: Integrating Synthetic Biology, Nanotechnology, and Systems Immunology for Improved Immunogenicity

**DOI:** 10.3390/vaccines13060588

**Published:** 2025-05-30

**Authors:** Majid Eslami, Bahram Fadaee Dowlat, Shayan Yaghmayee, Anoosha Habibian, Saeedeh Keshavarzi, Valentyn Oksenych, Ramtin Naderian

**Affiliations:** 1 Cancer Research Center, Semnan University of Medical Sciences, Semnan 35147-99442, Iran; majid.bac@gmail.com; 2 School of Medicine, Iran University of Medical Sciences, Tehran 14496-14535, Iran; 3 Nervous System Stem Cells Research Center, Semnan University of Medical Sciences, Semnan 35147-99442, Iran; 4 Clinical Research Development Unit, Kowsar Educational, Research and Therapeutic Hospital, Semnan University of Medical Sciences, Semnan 35147-99442, Iran; 5 Faculty of Medicine, University of Bergen, 5020 Bergen, Norway

**Keywords:** immunogenicity, immunoprophylaxis, next-generation platforms, vaccine, virus-like particles

## Abstract

The emergence of complex and rapidly evolving pathogens necessitates innovative vaccine platforms that move beyond traditional methods. This review explores the transformative potential of next-generation vaccine technologies, focusing on the combined use of synthetic biology, nanotechnology, and systems immunology. Synthetic biology provides modular tools for designing antigenic components with improved immunogenicity, as seen in mRNA, DNA, and peptide-based platforms featuring codon optimization and self-amplifying constructs. At the same time, nanotechnology enables precise antigen delivery and controlled immune activation through engineered nanoparticles such as lipid-based carriers, virus-like particles, and polymeric systems to improve stability, targeting, and dose efficiency. Systems immunology aids these advancements by analyzing immune responses through multi-omics data and computational modeling, which assists in antigen selection, immune profiling, and adjuvant optimization. This approach enhances both humoral and cellular immunity, solving challenges like antigen presentation, response durability, and vaccine personalization. Case studies on SARS-CoV-2, Epstein–Barr virus, and *Mycobacterium tuberculosis* highlight the practical application of these platforms. Despite promising progress, challenges include scalability, safety evaluation, and ethical concerns with data-driven vaccine designs. Ongoing interdisciplinary collaboration is crucial to fully develop these technologies for strong, adaptable, globally accessible vaccines. This review emphasizes next-generation vaccines as foundational for future immunoprophylaxis, especially against emerging infectious diseases and cancer immunotherapy.

## 1. The Evolution of Vaccines: From Traditional to Next-Generation Platforms

Vaccines are biological agents designed to stimulate the immune system, promoting the production of pathogen-specific antibodies and facilitating the development of active immunity [1]. There is a long history of vaccine development, representing one of the most important aspects of modern medicine. In this context, there is an observable path in vaccine development, initiated from live-attenuated forms and advancing to genetically engineered ones [2]. From another viewpoint, all vaccine history can be subdivided into only three generations [3].

Live attenuated vaccines were the first form produced; these were designed based on the ideas proposed by Jenner. He suggested that incubation with cowpox virus in healthy individuals may protect them against smallpox [4]. After that, Pasteur developed Jenner’s ideas about attenuation and designed three vaccines based on this idea, comprising the *Pasteurella multocida* vaccine (from a pathogen in chickens), the sheep anthrax vaccine, and a rabies vaccine. In the following years and because of the development of cell culture techniques, other types of live attenuated vaccines were also designed, including those for tuberculosis, yellow fever, polio, measles, mumps, rubella, and varicella [5]. While live-attenuated vaccines elicit robust, long-lasting humoral and cellular immune responses, their use is contraindicated in immunocompromised individuals and pregnant women, due to the potential risk of reversion to a virulent form, especially in immunocompromised individuals [6].

Frederick Griffith’s discovery in the 19th century paved the way for vaccine technology. Based on Griffith’s work and subsequent scientific studies, preserving immunogenicity is possible when bacteria are inactivated using controlled heat or chemical methods [7]. This was the key to the discovery of transforming processes, in which pathogen-like bacteria transform whole or partial parts (plasmid) of their genome into that of another. In this context, the inactivation of bacteria and the formulation of their components represent a new perspective in vaccine design. The first vaccine of this type was the typhoid vaccine, which led to other bacterial and viral vaccines encompassing those for cholera, pertussis, influenza, rabies, and hepatitis A [8].

The identification of polysaccharide capsules around certain pathogens led to the formulation of a novel type of vaccine that was not traditionally an inactivated form; these were known as conjugated vaccines because of the presence of a carrier molecule in association with the main antigen (which was the polysaccharide) [9]. This cluster comprises vaccines against *Neisseria meningitidis*, *Haemophilus influenzae*, and *Streptococcus pneumoniae*. Likewise, proteins could be purified and utilized for vaccine formulation. In this context, the protein was an antigen, stimulating the host’s immunity response [10,11]. The first vaccine of this type employed diphtheria toxoid [2]. Others, including anti-anthrax secreted proteins and anti-hepatitis B [12] vaccines, were developed later.

Advancements in genetic and molecular biology technologies led to the development of recombinant vaccines. Unlike subunit vaccines, which employ purified proteins, these types employ genes that produce antigens [13]. Following antigen selection, an expression system (such as yeast, mammalian cells, and bacteria) is used to produce the antigen in huge amounts. The hepatitis B vaccine is the best example of this family, which has exhibited remarkable success. The vaccine comprises a sequence of viruses that encode HbsAg, which is responsible for huge levels of antibody production. Other types of subunit vaccines are anti-HPV [14], anti-herpes, and anti-rotavirus vaccines [15].

Lessons from emerging infectious outbreaks such as influenza, Ebola, SARS-CoV-1, MERS, and SARS-CoV-2, alongside the likelihood of future pandemics arising from RNA viruses, underscore the growing need for innovative vaccine platforms. The rapid evolution of pathogens, their high transmission potency, and the adaptability of virulent strains are key concerns in the face of new outbreaks [16,17]. These challenges have driven the development of next-generation vaccine platforms, incorporating advancements like nanotechnology to enable rapid adaptation, strengthen immune responses, and optimize dosing regimens. Recent advancements in nanotechnology have introduced novel vaccine delivery systems, demonstrating the potential to enhance immune responses more effectively, even with lower or potentially single-dose regimens, a concept supported by experimental results that will be discussed in later sections [18]. As next-generation vaccine platforms evolve, integrating AI-driven antigen prediction and personalized immunoprofiling may further revolutionize preventive medicine, fostering rapid responses to future pandemics.

## 2. Synthetic Biology in Vaccine Design

Vaccines are one of the main components of public health, and they can reduce mortality and morbidity without causing severe illness, a process that requires two steps: (1) selecting the antigen, and (2) introducing it into the body [19]. Subunit vaccines, which deliver essential pathogen components, are a new trend in the vaccination industry. They are more common and safer than traditional whole-pathogen vaccines, but subunit vaccines are typically weak, they need multiple doses to be effective and must be combined with an adjuvant to enhance the immune response, while live attenuated viruses frequently elicit strong immune responses. In recent decades, subunit vaccines have been processed and/or chemically modified to develop synthetic vaccines with improved immunogenicity. Specifically, synthetic vaccine systems such as polymeric nanoparticles, inorganic nanoparticles, liposomes, and micelles have shown significant effects in enhancing vaccine efficacy [20].

Synthetic vaccines consist of molecular antigens linked to a carrier protein. This approach can reduce the issues associated with traditional vaccines, such as pathogenicity and the risk of infection caused by attenuated bacteria or viruses, or inactivated pathogens that are killed by heat or formaldehyde [21]. Nucleic acid vaccines based on DNA work either through cloned plasmids or directly via mRNA to express the antigen in host cells, and this novel therapeutic approach uses the cell’s machinery for protein synthesis to create the desired proteins that the given mRNA encodes. Protein synthesis in this context mimics natural infection, activating both the humoral and cell-mediated immune responses. This approach also enhances immunity and enables the rapid production of vaccines and customizable vaccines. Although the mRNA platform made rapid vaccine development possible, the need for very cold storage still limits its widespread use. Most resource-limited countries lack the cold-chain storage necessary to carry out mass vaccinations [22,23,24]. During the SARS-CoV-2 pandemic, some mRNA vaccines, such as Moderna’s mRNA-1273 and BNT 162b2, were improved by Pfizer-BioNTech in 2020. COVID-19 mRNA vaccines have become the first FDA-approved mRNA vaccination for human use [25].

The production of proteins without the requirement for cell culture systems significantly reduces manufacturing costs. mRNA vaccines are generated through in vitro transcription using enzymatic reactions, although DNA serves as the initial template. The plasmid DNA required for this process is replicated in *Escherichia coli*. DNA-based vaccines exhibit greater stability compared to mRNA vaccines, thereby eliminating the need for ultra-low temperature storage and specialized cold-chain logistics. However, a recurring theme in human DNA vaccine trials has been their suboptimal immunogenicity when compared to traditional protein-based vaccine approaches. If they are to be used in humans, methods to improve the immunogenicity of DNA vaccines must be developed. These methods include electroporation, the co-expression of plasmids encoding adjuvants like cytokine-encoding genes, the co-formulation of DNA vaccines with conventional adjuvant compounds, codon optimization to maximize protein expression, the boosting of DNA vaccines with live viral vectors, or adjuvanted protein vaccines [26,27].

**Figure 1:**
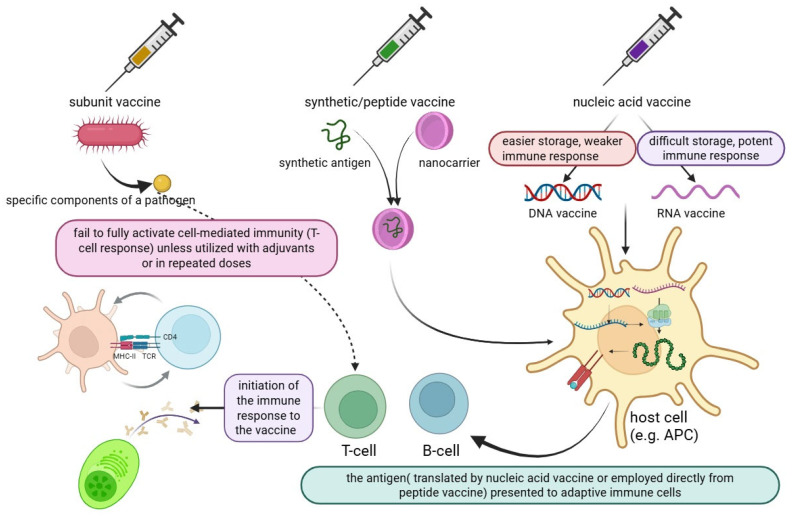
An overview of the three main novel vaccine technologies, synthetic biology, nanotechnology, and systems immunology, along with a depiction of their underlying molecular mechanisms and points of interaction.

**Figure 2:**
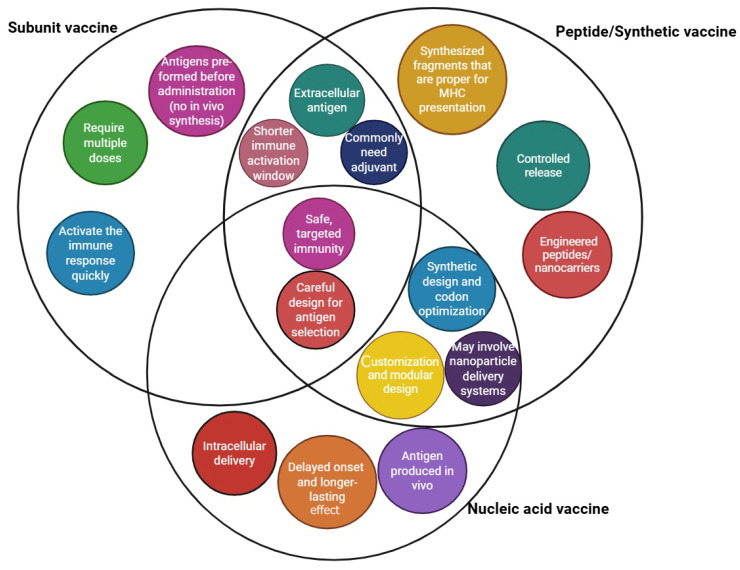
Summary of peptide, nucleic acid, and synthetic vaccine technologies in the context of their unique and common characteristics.

Peptide vaccines are synthesized using fragment condensation methods, and these vaccines are typically administered with an adjuvant to enhance the immune response. Additionally, these vaccines are considered safer than live or attenuated vaccines [28]. As biotechnology has advanced quickly, recombinant proteins for application in vaccines and medications have been produced by heterologous expression. As the fundamental unit of correspondence between information-carrying proteins and nucleic acids, the codon serves as the fundamental conduit for information transfer in vivo. Known as synonymous codons, they encode the same amino acid. Codon usage bias refers to the tendency of a species or a gene to favor the use of one or more specific synonymous codons, known as optimum codons, even when the usage probability of synonymous codons is not the same during protein synthesis. Additionally, the bias governing codon use by genes varies greatly, depending on the function. This phenomenon is called codon usage bias. Heterologous expression has been used to produce recombinant proteins for application in medications and vaccines, as biotechnology has advanced quickly, even though the usage probability of synonymous codons is not the same during protein synthesis [29,30]. There are also other advantages to employing the attenuation technique of codon deoptimization. A successful response to infectious epidemics requires speed, and this method has the advantage of not requiring an in-depth knowledge of viral function. The three novel aspects of vaccine technology are illustrated in Figure 1. Additionally, a summary of these three technologies is provided in a diagram highlighting their exclusive and shared features, as shown in Figure 2.

Computational methods make it possible to characterize codon biases and forecast protein-coding areas using genomic data [31,32]. Because of the hundreds of mutations, virulent reversion is extremely uncommon, and a single dose may be enough to produce long-lasting protective immunity, making deployment easier. Furthermore, the genes’ codon use bias varies greatly, depending on their function. Codon deoptimization vaccines have been evaluated in several phase I clinical trials, including CodaVax-H1N1 (for influenza A H1N1), CodaVax-RSV (for respiratory syncytial virus), and CDX-005 (for SARS-CoV-2), with clinical trials for the latter planned for early 2021 [33].

### 2.1. Integrated Perspective on Vaccine Adjuvants: Common Mechanisms and Strategic Insights

Adjuvants are defined as different substances that, when given with vaccination antigens, increase the immunogenicity of vaccines [34]. Adjuvants can be anything from complicated natural extracts and particulate matter to tiny artificial molecules. Alexander Glenny discovered, in 1926, that injecting aluminum salts with antigens into guinea pigs produced more antibodies than injecting antigens alone, providing the first proof of adjuvants. Freund and his associates subsequently created water-in-oil emulsions in the 1940s, which resulted in the development of Freund’s adjuvants [35]. However, due to its toxicity to people, Freund’s adjuvant is not authorized for use in human vaccinations. Like Freund’s adjuvants, bacterial lipopolysaccharide (LPS) adjuvants have had limited usage in human vaccines because of both their systemic and local adverse effects. Aluminum adjuvants are the most common mineral salts. For more than 60 years, many nations have used aluminum adjuvants in their regular childhood vaccination regimens, mainly in tetanus, diphtheria, pertussis, and poliomyelitis vaccines. Aluminum adjuvants were then added to vaccines for use against the hepatitis A and hepatitis B viruses, the human papillomavirus (which causes cervical cancer and genital warts), Lyme disease/Borreliose, and Japanese encephalitis. For specific risk populations, additional aluminum-adsorbed vaccines are available to prevent diseases like anthrax. Emulsions are another type of adjuvant. Both AS03 and MF59 are traditional adjuvants for oil-in-water emulsions [35].

The first non-aluminum adjuvant authorized for use in human vaccinations was MF59, which was licensed in 1997 as an adjuvant for the influenza vaccine. The two functions of MF59 emulsions are immune activation and antigen delivery. By gradually releasing antigens into the lymph nodes, MF59 can be employed as an emulsion delivery method in conjunction with antigens to enhance antigen presentation and prolong antigen engagement with the immune system [36,37]. Despite attempts to create new adjuvants for human vaccinations, only aluminum adjuvants were licensed between the 1920s and the 1990s. The oil-in-water emulsion MF59 was not approved for use as an adjuvant in influenza vaccines in Europe until 1997. The monotony of adjuvants for human vaccinations was broken in the next two decades with the licensing of four additional adjuvants (AS04, AS03, AS01, and CpG ODN 1018) for use in vaccines [38]. During this period, numerous other kinds of chemicals, such as mineral salts, microbial products, emulsions, saponins, synthetic small molecule agonists, polymers, nanoparticles, and liposomes, were also assessed as adjuvants [39]. Preclinical and clinical research has demonstrated that they improve the robustness, range, and durability of immune responses [40]. Adjuvants function through two mechanisms: delivery systems and immunostimulants. By targeting particular pattern-recognition receptors (PRRs), immunostimulants stimulate the antigen-presenting cells (APCs), resulting in improved antigen-presenting and co-stimulatory signaling and an improvement in the adaptive immune system. By extending the bioavailability of antigens, the delivery systems direct the antigens to APCs or lymph nodes, improving the absorption and presentation of antigens by APCs and boosting the adaptive immune response. In order to help with the selection of sensible adjuvants during the development of a particular vaccine, this review will go into detail on both traditional adjuvant platforms and the adjuvant platforms that are currently being studied. We believe that the following issues might need to be addressed in order to encourage the more widespread development of adjuvants [35]. Clinically approved adjuvants collectively exhibit common mechanisms that are capable of increasing immunogenicity. Most adjuvants achieve this either by activating innate immunity via PRRs, as seen with CpG 1018 and AS01, or by establishing an antigen depot effect, as exemplified by alum and MF59, which extends antigen availability to bolster adaptive immune responses. Additionally, emulsions such as AS03 and MF59 promote antigen uptake and the recruitment of APCs, while Toll-like receptor (TLR) agonists like CpG actively drive dendritic cell maturation. Notably, Th1/Th2 polarization varies significantly among adjuvants: alum primarily induces a Th2-biased response, while AS01 and CpG favor Th1 responses, which are especially essential for addressing intracellular pathogens and cancer. This comparative analysis highlights the importance of strategically selecting adjuvants that align with the pathogen type, desired immune response, and characteristics of the target patient population (Table 1).

### 2.2. Key Functional Characteristics of Vaccine Adjuvants

Self-amplifying RNA (saRNA) and standard mRNA are the two forms of synthetic RNA vaccines that are currently available (see Figure 1). Numerous preclinical and clinical studies have examined the use of conventional mRNA strategies—also known as nonreplicating or non-amplifying mRNA—against cancer and infectious disorders. While these encoding therapeutic proteins, including antibodies or immune modulators, have been examined for immunotherapy, in vitro transcribed mRNAs encoding viral antigens have been investigated as vaccines [41,42]. Nonetheless, the quantity of conventional mRNA transcripts that are successfully administered during immunization is directly correlated with antigen expression. Therefore, high dosages or repeated administrations may be necessary to achieve sufficient expression for immunomodulation or protection. Genetically modified replicons generated from self-replicating single-stranded RNA viruses are known as saRNA vaccines. They can be administered as fully synthetic saRNA, generated following in vitro transcription, or delivered as viral replication particles (VRPs) with the saRNA enclosed within the viral particle. During manufacturing, envelope proteins are supplied in the form of faulty assistance constructions to produce replication-defective VRPs. After an initial infection, the resulting VRPs are unable to produce infectious viral particles, and only the RNA can be amplified further. Both positive-sense and negative-sense RNA viruses can produce VRPs, although the latter are more complicated and need reverse genetics to be saved (Table 2).

## 3. Nanoparticle Properties and Their Immunological Impacts

### 3.1. Size

The size of nanoparticles plays a crucial role in their uptake by APCs and the subsequent activation of the immune system. Particles within the 20–200 nm range are efficiently internalized by dendritic cells and macrophages, enabling effective antigen processing and presentation. Smaller nanoparticles, which are typically less than 50 nm, can migrate into the lymph nodes, enhancing interactions with resident immune cells. In contrast, larger particles, those exceeding 500 nm, are more likely to be involved in phagocytosis by macrophages at the injection site.

### 3.2. Shape

Nanoparticle shape significantly influences cellular uptake and distribution throughout the body. Spherical nanoparticles are generally internalized more easily compared to rod-shaped or irregular forms. However, rod-shaped nanoparticles benefit from prolonged circulation times and offer improved tumor penetration, making them particularly valuable in cancer immunotherapy.

### 3.3. Surface Charge

The surface charge of nanoparticles impacts their interaction with cell membranes and proteins. Positively charged nanoparticles show increased cellular uptake due to electrostatic attraction to negatively charged cell membranes, although this can also lead to higher cytotoxicity and nonspecific binding. In contrast, negatively charged or neutral nanoparticles tend to reduce nonspecific interactions and exhibit longer circulation times.

### 3.4. Composition

The composition of nanoparticles determines factors such as biodegradability, antigen release dynamics, and intrinsic immunostimulatory properties. For example, lipid-based nanoparticles excel at facilitating endosomal escape, promoting cytosolic antigen delivery, while polymer-based nanoparticles can be tailored for controlled release and targeted delivery [49].

### 3.5. Molecular and Cellular Mechanisms

Nanoparticles boost immune responses through an interconnected network of molecular and cellular pathways.

Enhanced antigen presentation: Nanoparticles improve the precise delivery of antigens to APCs, such as dendritic cells and macrophages. This facilitates efficient processing and presentation through major histocompatibility complex (MHC) class I and II pathways, triggering the strong activation of CD4+ and CD8+ T cells.

Activation of innate immunity: Certain nanoparticle formulations can interact with PRRs, including TLRs and NOD-like receptors (NLRs). This interaction induces the production of proinflammatory cytokines, such as IL-6 and TNF-α, along with the upregulation of co-stimulatory molecules. Such innate immune activation plays a fundamental role in shaping effective adaptive immune responses.

Modulation of immune cell trafficking: By engineering nanoparticles with specific surface ligands or optimizing their size, it becomes possible to target particular tissues or subsets of immune cells. These tailored particles can influence immune cell migration, retention, and activation, particularly within the lymphoid tissues or areas of inflammation [50,51].

## 4. Nanotechnology as a Vaccine Delivery System

Recent advancements in nanotechnology have introduced novel vaccine delivery systems, demonstrating the potential to enhance immune responses more effectively, even with lower or potentially single-dose regimens. Nanoparticles (NPs) encompass a diverse class of materials and are characterized by a diameter of less than 100 nm; their unique physicochemical properties render them highly suitable for biomedical applications. Beyond their established potential for drug delivery and controlled release, nanoparticles can also exhibit intrinsic antigenic properties, sparking considerable scientific interest in harnessing this technology as an innovative vaccine platform [18]. Nanovaccines offer a targeted tissue delivery system, which represents one of their key advantages. Their structural composition protects vaccine antigens from enzymatic degradation and enhances antigen transport to the lymphatic system, improving immune activation. Conventional peptide-based vaccines often fail to induce cross-presentation by dendritic cells (DCs), limiting CD8+ T cell activation. Nanovaccines overcome this limitation by facilitating efficient antigen presentation through both the MHC I and MHC II pathways, promoting CD8+ T cell activation and enhancing CD4+ T cell responses, ultimately supporting B cell activation and antibody production. Moreover, nanovaccine adjuvants can engage pattern-recognition receptors on DCs, driving DC maturation, type I interferon expression, and antiviral cytokine production. Their enhanced delivery efficiency, superior DC activation, sustainable antigen release, and ability to present high antigen concentrations for optimal B cell receptor binding make nanovaccines promising candidates for inducing robust, long-lasting antibody responses [35,52]. LNPs, polymer-based nanoparticles, protein-based nanoparticles, metal-based nanoparticles, virus-like particles (VLPs), and liposomes are examples of nano-based vaccine platforms [18,53]. In a study evaluating an Epstein–Barr virus (EBV) vaccine, protein-based polymeric NPs were co-formulated with EBV glycoprotein B (gB) to assess immunogenicity in BALB/c mice. Control groups received either EBV gB or nanoparticles alone. To investigate the adjuvant effects, oil-in-water emulsion MF59 and Imject Alum were incorporated. Mice received three doses at weeks 0, 3, and 8. The results demonstrated that the gB-NP formulation elicited higher neutralizing antibody titers in epithelial cells compared to B cells [54].

Notably, anti-gB IgG titers were highest in the gB-NP group adjuvanted with MF59, maintaining elevated levels through week 20 that were comparable to the peak titers observed at week 10. The candidate vaccine was further evaluated in *Macaca fascicularis* as a non-human primate (NHP) model. Immunogenicity analysis revealed that total anti-gB IgG (*p* < 0.01) and anti-gB IgA titers (*p* < 0.01), along with B cell and epithelial cell neutralization titers (*p* < 0.0001), were significantly higher in the gB-NP vaccine group compared to the gB-alone formulation, with marked superiority in terms of epithelial cell neutralization. Moreover, the passive transfer of serum from vaccinated NHPs to humanized mice conferred protection against an EBV challenge, demonstrating the functional efficacy of the induced immune response [54].

A recent study introduced the vaccine delivery system X (VADEX), an innovative self-adjuvanted protein nanoparticle platform designed to simultaneously enhance antigen presentation and immune stimulation within a single molecular framework. The VADEX system is built on a fusion protein comprising an amphipathic helical peptide and a superfolder green fluorescent protein (sfGFP) that self-assembles into stable nanoparticles. This structural configuration not only supports multivalent antigen display but also provides intrinsic immunostimulatory properties, eliminating the requirement for external adjuvants. A key finding of the study emphasized the significance of the amphipathic helical peptide for maintaining the nanoparticle’s thermal stability and structural integrity, both of which are essential for ensuring vaccine stability during storage and distribution. By employing split-GFP technology, the researchers analyzed the impact of peptide sequence integrity and thermal resilience. Their results revealed that the VADEX platform exhibited superior self-adjuvanticity when compared to a clinical-stage adjuvant, as demonstrated by improved antibody binding affinity and a higher-quality immune response. Generally, VADEX marks a significant advancement in protein-based vaccine platforms, delivering a streamlined, stable, and highly effective method for inducing strong humoral and cellular immune responses without the need for additional adjuvants. The principles underlying its design could also be applied to therapeutic antibody development and other fields where robust immune activation is essential [55].

Furthermore, H1ssF recipients exhibited a fivefold rise in H5 stem-specific antibody titers by week 2, which persisted through week 16. Following the booster dose, the titers increased to eightfold by week 18 and remained elevated through week 40. Neutralizing antibodies against H5N1 similarly increased after both the prime and booster doses, sustaining significant elevation at week 40. A comparable trend was observed for H2N2 stem-binding and neutralizing antibodies, indicating broad cross-reactivity [56]. The first nano-based vaccine platform targeting *Coxiella burnetii*, the causative agent of Q fever, utilized an outer membrane protein antigen (CBU1910) conjugated to an E2 protein-based nanoparticle through three distinct methods: (1) the direct fusion of CBU1910 to E2, (2) Maleimide-tNTA-Ni linker chemistry using cysteine-rich E2 with His-tagged CBU1910, and (3) the SpyTag/SpyCatcher system for conjugating ST-E2 and SC-CBU1910. Among these, the third approach demonstrated superior physical stability and antigen-loading capacity. Notably, this formulation induced over ninefold higher IgG antibody levels compared to using the soluble antigen alone—even without an adjuvant. However, the response was skewed predominantly toward IgG1 (a Th2 response marker), with an imbalance in IgG2 (a Th1 response marker). This imbalance was corrected by incorporating CpG1826 as an adjuvant, leading to a more balanced Th1/Th2 response profile. Despite robust humoral immunity, antigen-specific IFN-γ production—a key marker of cellular immune activation—remained insufficient. This limitation was resolved by adding IVAX, which enhanced the cellular immune response. Collectively, these findings highlight the potential of nano-based vaccine platforms for *C. burnetii* and emphasize the need for further experimental optimization [57]. A recent study has developed a bionic nanovaccine that incorporates self-adjuvant properties using macrophage-derived vesicles co-loaded with antigens from the monkeypox virus. This innovative approach demonstrated strong antigen presentation capabilities and improved immune protection in preclinical models [58].

In another study, *Mycobacterium tuberculosis* (Mtb) antigens were first engineered as protein fusions with an N-terminal histidine tag and heparin-binding hemagglutinin adhesin, then they were loaded onto yellow carnauba palm wax nanoparticles with sodium myristate (YC-NaMA NPs). Two intranasal booster doses of Nano-FP1, administered at weeks 2 and 11, significantly enhanced short-term lung protection against Mtb infection compared to BCG alone. Additionally, the first booster dose increased the numbers of CD4+ resident memory T cells and T cells producing IFN-γ, TNF-α, IL-17, or combinations of IFN-γ+ TNF-α+ and IFN-γ+ TNF-α+ IL-2+, alongside an expansion in the CD8+ T cell population—although this response waned after 7 weeks [59]. In another study, the CV0501 vaccine, which is composed of SARS-CoV-2 Omicron BA.1 variant mRNA encapsulated in LNPs, was evaluated across five cohorts of up to 30 participants receiving 12, 25, 50, 100, or 200 μg doses. Participants had previously been vaccinated and were monitored for 6 months. Lower doses (3 and 6 μg, each administered to two groups of 15 participants) were subsequently evaluated after safety and immunogenicity assessments on day 29. Injection-site pain was the most frequently reported adverse event (57.5%, *n* = 180), followed by myalgia (36.9%) and fatigue (33%) [60].

Three serious adverse events (SAEs), namely, cholecystitis, diarrhea, and syncope, were reported but were determined to be unrelated to the vaccine. The neutralizing antibody titers peaked on day 15 and remained significantly elevated compared to pre-boost levels on day 181. Geometric mean titers (GMTs) were highest against the Omicron BA.1 subvariant, with cross-reactive responses observed against BA.2, BA.5, Delta, and wild-type strains. A dose-response relationship was evident, with higher doses generating correspondingly higher antibody titers. Cellular immunity analysis revealed a rise in polypositive CD4+ T cell frequencies by day 8, plateauing on day 15 against the wild-type, BA.1, and BA.5 subvariants. Notably, CD4+ T cells exhibited increased IFN-γ expression, which was indicative of a Th1-skewed immune response, while IL-13 and IL-17 expression remained unchanged. In contrast, no significant changes from baseline were observed in polypositive CD8+ T cell responses [60]. Another study on *Listeria monocytogenes* identified LMON_0149, a periplasmic oligopeptide-binding protein (OppA), as a promising vaccine target. Following a proteomics-based analysis, LMON_0149 was selected for an mRNA vaccine that was formulated using a lipid nanoparticle (LNP) platform. The vaccine demonstrated a significant induction of antigen-specific IFN-γ expression in mice, marking a strong CD8+ T cell response [61]. In another study on influenza and SARS-CoV-2, researchers encoded the Chemokine (C-X-C motif) ligand 13 (CXCL13) within antigen-encoding circular RNA (circRNA) strands—hemagglutinin (HA) for influenza and trimeric receptor-binding domain (RBD) for SARS-CoV-2—which were delivered via LNPs. The results demonstrated that HA-CXCL13–circRNA induced a threefold increase in germinal center B cell numbers compared to HA–circRNA in mice. Notably, while germinal center B cell numbers declined in the HA–circRNA group after day 14, they remained stable in the HA-CXCL13–circRNA group. Additionally, HA-CXCL13–circRNA elicited a stronger CD4+ and CD8+ T cell response, characterized by elevated IL-2 and TNF-α expression rather than IFN-γ. Moreover, cross-reactive antibody responses were significantly enhanced in the HA-CXCL13–circRNA group. Mice primed with an HA sequence from H3N2 and later challenged with PR8 (H1N1) exhibited 100% survival in the HA-CXCL13–circRNA group, whereas 4 out of 10 mice succumbed in the HA–circRNA group. Similarly, when comparing RBD-CXCL13–circRNA with RBD–circRNA, the former induced significantly higher antibody titers against the Wuhan-Hu-1 S protein, S1 protein, RBD protein, and multiple RBD mutants, while the latter showed markedly lower responses. These findings suggest that CXCL13 may enhance cross-reactivity in COVID-19 vaccines as well [62]. Metal-based nanoparticles, primarily of gold and iron oxides, are commonly employed as adjuvants within vaccine platforms [63]. However, a meta-analysis of animal studies indicated that these nanoparticles did not demonstrate superior efficacy compared to conventional adjuvants or antigen-alone formulations. Notably, 75% of the reviewed studies utilized gold nanoparticles as the adjuvant of choice [64].

Liposomes are bilayer structures that are primarily composed of phospholipids surrounding an aqueous core, making them highly biocompatible and, thus, an attractive candidate for vaccine delivery systems. While liposome-based vaccines have demonstrated promising results, their susceptibility to enzymatic degradation, pH fluctuations, and immune system interference limits their stability and effectiveness compared to LNPs [18]. Another study evaluated a *Plasmodium falciparum* full-length recombinant Circumsporozoite (rCSP) vaccine that was combined with glucopyranosyl lipid A–liposome Quillaja saponaria 21 formulation (GLA-LSQ), a nanoliposomal adjuvant, reporting that it demonstrated a favorable safety profile with no SAEs. However, the vaccine failed to induce protective immunity. Notably, higher doses, with 60 µg as the highest dose, elicited increased IgG titers, indicating the potential for dose optimization and the need for further investigation to enhance immunogenicity [65]. Another study investigated a *Chlamydia trachomatis* vaccine combining a recombinant major outer membrane protein (CTH522) with cationic liposomal adjuvants, CAF01 and CAF09b, comparing these formulations against each other and a placebo group. Intramuscular (IM), intradermal, and topical delivery methods were also evaluated. No SAEs were reported. IgG anti-CTH522 titers were highest in the 85 µg group, compared to the 15 µg group. However, no significant differences were observed between the 85 µg IM group, which received booster doses on days 28 and 112 with mucosal recall boosters at day 140, with either a placebo or topical vaccine using CAF01 or CAF09b. Intradermal CTH522 delivery induced robust neutralizing antibody responses against serovars B (trachoma) and D (urogenital infections) [66].

Additionally, topical ocular administration of the vaccine resulted in elevated IgA titers compared to baseline. Cell-mediated immune responses were detected across all active treatment groups [66]. VLPs are self-assembling structures composed of one or more viral proteins, organized symmetrically within a diameter range of 20 to 120 nm, mimicking the morphology of native virus particles. Their intrinsic adaptability to genetic and chemical modifications positions them as highly versatile antigen delivery platforms that are capable of incorporating multiple antigenic epitopes. This multi-epitope presentation enhances their potential for the broad-spectrum detection of diverse viral agents within a single assay format, offering a promising approach for multiplex vaccine applications [67]. In a recent study, the full-length SARS-CoV-2 spike protein was successfully expressed in *Nicotiana benthamiana*, purified, and subsequently formulated with the AS03 adjuvant. Healthy participants were randomized into nine groups, receiving CoVPL at doses of 3.75, 7.5, or 15 µg, combined with CpG 1018 or AS03, or administered without an adjuvant. Immunogenicity assessments from day 1 to day 42 demonstrated that AS03-adjuvanted formulations across all CoVPL doses elicited significantly higher and more durable anti-spike IgG and live virus-neutralizing antibody (nAb) responses, which are key indicators of humoral immunity [68]. Additionally, CoVPL used alone induced sustained IFN-γ and IL-4 responses after the second dose, whereas CoVPL with AS03 produced more robust cellular immunogenicity [68]. Long-term data collected on days 201 and 386 revealed that participants receiving CoVPL 3.75 µg + AS03 exhibited a significant decline in anti-spike IgG and live virus-neutralizing antibody titers at day 201 compared to day 42; however, these titers remained notably higher than those observed at baseline (day 21) [69].

Similarly, spike-specific IFN-γ and IL-4 responses decreased by day 201 relative to day 42 but remained elevated compared to day 21 levels. Importantly, no severe adverse events (SAEs), withdrawals, or fatalities were reported during the study, with only mild to moderate adverse events observed, alongside a single case of grade-three fatigue [69]. These findings suggest that a two-dose regimen of CoVPL + AS03, administered three weeks apart, effectively induces both cellular and humoral immunity against ancestral SARS-CoV-2 strains, supporting its potential as a viable vaccine candidate. In another study, a nanovaccine incorporating the pan-epitope peptide TBT, combined with CpG, was tested in mice to evaluate its immunogenicity against flaviviruses. The results demonstrated that bone marrow-derived DCs exhibited a higher uptake rate of TBT − CpG compared to TBT alone or TBT + CpG, leading to an increased expression of proinflammatory cytokines such as IL-1β, IL-6, and TNF-α. Moreover, antigen-specific IgG production was observed, along with significantly elevated IL-4 (*p* < 0.001) and IFN-γ (*p* < 0.01) expression in spleen cells. Survival analysis revealed that 20% (*p* < 0.01) of three-day-old mice receiving TBT-CpG and challenged with DENV-2 or DENV-4 survived beyond 15 days, while a higher survival rate of 35% (*p* < 0.001) was recorded in one-day-old, immunized mice challenged with ZIKV (19). Notably, levels of alanine aminotransferase (ALT) and aspartate aminotransferase (AST) remained within the normal range in immunized mice, suggesting the vaccine’s protective potential against Dengue virus (DENV) and Zika virus (ZIKV) infections [70].

In a first-in-human study, an enveloped virus-like particle (eVLP) vaccine targeting cytomegalovirus (CMV) was evaluated across a three-dose schedule (days 0, 56, and 168), with immunogenicity assessments being conducted on fibroblast and epithelial cell models. Participants received 0.5, 1, or 2 µg of glycoprotein B (gB) formulated with alum, 1 µg without alum, or a placebo. The most commonly reported local and systemic adverse events (AEs) were mild pain (without any associated swelling or erythema) and headache, both of which remained consistent in frequency and severity across the dosing groups. The serological analysis demonstrated that anti-CMV gB antibody titers and avidity were highest in the cohort receiving 2 µg with alum. Although antibody titers declined by 20–30% across all groups over time, the levels remained notably above baseline. Neutralizing antibody (nAb) responses demonstrated robust activity in fibroblasts, while responses in the epithelial cells were more modest, indicating the need for further optimization to enhance cross-cellular immunity [71]. Recent advancements in nanotechnology have significantly transformed vaccine development, offering innovative strategies to enhance immunogenicity, antigen stability, and targeted delivery. The studies reviewed highlight the diverse applications of nanovaccine platforms, including lipid-based nanoparticles, polymeric nanoparticles, virus-like particles, and metal-based nanoparticles, each demonstrating unique advantages in terms of antigen presentation, immune system activation, and cross-reactivity enhancement. Notably, nanovaccines facilitate both MHC I and MHC II antigen presentation, enhancing CD8+ T cell activation and supporting robust B cell-mediated antibody production. This property is particularly valuable for addressing the challenges associated with conventional peptide-based vaccines that fail to elicit strong cytotoxic responses. Furthermore, the use of nano-based adjuvants, such as CpG and CXCL13-conjugated platforms, underscores their potential to modulate immune responses toward a more balanced Th1/Th2 profile, a critical factor in effective and durable immunity. Preclinical and clinical evaluations have demonstrated promising immunogenicity and safety profiles across various infectious disease models, including Epstein–Barr virus, influenza, *Mycobacterium tuberculosis*, SARS-CoV-2, and *Coxiella burnetii*. Additionally, studies incorporating novel delivery mechanisms, such as circular RNA encoding immunomodulatory factors, suggest new avenues for the further optimization of vaccine efficacy. Despite these promising findings, several challenges remain, including the need for standardized formulations, long-term safety assessments, and large-scale production capabilities. Additionally, the immunogenic variability among different nanovaccine platforms underscores the necessity for further comparative studies to identify the most effective approaches for specific pathogens. Future research should focus on refining antigen delivery mechanisms, optimizing dose regimens, and leveraging artificial intelligence-driven models to predict immune responses and enhance vaccine design. Collectively, the evidence presented herein reinforces the transformative potential of nanovaccine platforms in advancing next-generation immunization strategies. With continued innovation and rigorous clinical validation, nano-based vaccines may redefine prophylactic and therapeutic approaches to infectious diseases, addressing global health challenges with greater precision and efficacy. Nanoparticle platforms exhibit several common functional advantages. Nanovaccines reliably boost antigen stability and ensure precise delivery to lymphoid tissues, addressing the shortcomings of traditional peptide vaccines. They support dual MHC class I and II presentation, increasing CD8+ cytotoxic and CD4+ helper T cell responses. Moreover, their pathogen-like structures and customizable surfaces facilitate multivalent antigen presentation and the direct activation of B cells. These attributes position nanoparticles as optimal frameworks for designing advanced vaccines that promote robust, durable, and broad-ranging immunity (Table 3). 

## 5. Systems Immunology: Decoding Immune Responses

The integration of computational modeling with multi-omics data, including transcriptomics and proteomics, to analyze and interpret immune system functions is referred to as systems immunology [72]. Transcriptomics is the study of all biological aspects of ribonucleic acids (RNA), including their structure, transcription, translation, and cellular functions [73]. Proteomics, on the other hand, focuses on investigating protein structures and functions, with mass spectrometry serving as a cornerstone technology [74]. Together, these disciplines form a powerful, integrated approach that enables holistic and quantitative insights into molecular interactions, facilitating the prediction of biological responses and enhancing therapeutic performance [75]. The immune response to threats involves various subsets of leukocytes, categorized into innate and adaptive immune responses. The primary distinction lies in the adaptive response’s reliance on antigen-specific receptors and direct antigen interactions with T and B cells. While immune cell dynamics and cytokine signaling are essential, this discussion focuses specifically on antigen recognition and receptor interactions, given their critical role in vaccine function. Monocytes and macrophages act as phagocytes, processing microbial antigens through proteolysis to generate peptide fragments. These fragments are then presented to T cells via their T cell receptors (TCRs), contributing to T cell activation [76].

Among APCs, dendritic cells (DCs) are the most potent. They are widely distributed across tissues, with a high concentration in secondary lymphoid organs, where they facilitate antigen recognition through MHC class I and II molecules interacting with TCRs a key step in initiating adaptive immunity. B cells, alongside atypical APCs such as mast cells, eosinophils, basophils, epithelial cells, endothelial cells, and innate lymphoid cells (ILCs), have also demonstrated antigen-presenting capabilities [76,77,78]. MHC class I interactions arise from endogenous antigen processing, where intracellular proteins undergo proteasomal degradation, triggered by IFN-γ, yielding peptide fragments that bind to MHC class I molecules. This process is essential for activating CD8+ cytotoxic T cells, enabling the targeted destruction of infected or malignant cells [76]. Interestingly, cross-presentation, where exogenous antigens are presented on MHC class I molecules, plays a pivotal role in antiviral immunity. This mechanism can bypass the traditional endogenous antigen pathway, offering a strategic advantage in terms of immune response modulation. In contrast, MHC class II interactions stem from the exogenous antigen presentation pathway. Antigens are internalized via endocytosis or phagocytosis and processed into peptide fragments. This pathway, which can also be induced by IFN-γ in the epithelial and endothelial cells of an inflammation site, leads to the activation of CD4+ T helper cells, orchestrating broader immune responses, including B cell activation and cytokine signaling. Co-stimulatory signaling, through CD28 on T cells binding to CD80 and CD86 on APCs, plays a crucial role in achieving full T cell activation. Without this interaction, T cells may enter an anergic state, rendering them unresponsive [76].

Regulatory T cells (Tregs) are essential for modulating immune responses and maintaining tolerance. They can express both CD4 and CD8 markers and are classified into two subsets: natural Tregs (nTregs) and adaptive (induced) Tregs (iTregs). nTregs develop in the thymus and are characterized by the expression of CD4, CD25, and forkhead box protein 3 (Foxp3), a transcription factor that is critical for their development and function. In contrast, iTregs differentiate from naive CD4+ T cells in the peripheral tissues upon encountering specific antigens, with their formation influenced by IL-10 concentration during the initial phase. Both subsets secrete IL-10 and TGF-β to regulate immune activity. Resting naive CD4+ T cells, or T helper (Th) cells—those that have not yet differentiated into functional subtypes—produce IL-2 and can polarize into distinct effector subsets, based on the cytokine environment at the activation site [76].

Key differentiation pathways include Th1 cells, which are driven by IL-12, express the transcription factor T-box expressed in T cells (T-bet), and produce IL-2, IFN-γ, and lymphotoxin. They are primarily involved in cellular immunity, promoting macrophage activation and cytotoxic responses. Th2 cells, induced by IL-4, express the transcription factor GATA3 and produce IL-4, IL-5, IL-6, IL-9, IL-13, and GM-CSF. They support humoral immunity, driving B cell differentiation and antibody production [76,79]. Th17 cells, promoted by IL-6 (synergistically with IL-1β [80]) and TGF-β, express retinoic acid-related orphan receptor C isoform 2 (RORC2) and produce IL-6, IL-17, and other proinflammatory cytokines. They are crucial for defense against extracellular pathogens and play a role in autoimmune responses. The Th1 and Th2 subsets represent distinct immune pathways, with Th1 driving cellular immunity and Th2 supporting humoral immunity. B cells can be activated through both T cell-dependent and T cell-independent pathways. The T cell-dependent pathway, which generates a stronger and longer-lasting memory, involves B cells acting as APCs. This leads to MHC class II, CD80, and CD86 expression. Co-stimulation between the CD40 ligand on T cells and CD40 on B cells then drives antibody class switching and somatic mutation. In the T cell-independent pathway, B cells are activated by polymeric antigens, likely through cross-linking and the clustering of immunoglobulins on the B cell surface [76].

Some studies suggest that nanoparticle delivery systems may also directly activate B cells, contributing to optimal antibody responses. Numerous studies have shown that vaccine adjuvants stimulate IL-1 family, IL-6, IL-10, IL-12, and TNF-α cytokines upon reaching the injection site by activating APCs [35,80]. IL-1α, produced by endothelial cells, epithelial cells, monocytes, macrophages, DCs, and B lymphocytes [81], and IL-1β, produced by monocytes, macrophages, and DCs [81], are potent activators of conventional DCs and Langerhans cells (LCs), which are specialized phagocytes in the epidermis. LCs can promote naïve CD4+ T cell polarization into Th2 and Th17 subsets, alongside the priming and cross-priming of CD8+ cytotoxic T cells. Additionally, IL-1α enhances monocyte-derived DCs, boosting IFN-γ and IL-13 production, while IL-1β supports CD40L-mediated DC activation, leading to increased IFN-γ production in T cells and IL-12 secretion. Moreover, IL-1β directly boosts antigen-specific CD8+ T cell function, enhancing granzyme B and IFN-γ expression, as well as improving their migration and survival. IL-18, produced by blood monocytes, intestinal epithelial cells, and human LCs, promotes both conventional and plasmacytoid DC, which are specialized producers of type I interferons (IFN-I) and key mediators of antiviral immunity, in terms of migration to the lymph nodes. In synergy with IL-12 and IL-1β, IL-18 plays a key role in Th1 polarization and further upregulates IFN-γ expression [80]. IL-6 influences monocyte differentiation, promoting macrophage development in the presence of macrophage colony-stimulating factor (M-CSF) at high concentrations, while encouraging DC generation under high granulocyte-macrophage colony-stimulating factor (GM-CSF) and TNF-α conditions. It plays a pivotal role in DC maturation, indirectly supporting maturation by synergizing with IFN-γ or by counteracting TGF-β’s inhibitory effects, while also directly inhibiting maturation [82].

Furthermore, IL-6 supports DC migration to the lymph nodes or skin, suppresses regulatory T cell (Treg) activity, and promotes CD4+ T cell expansion in non-tumor environments [82]. It also stimulates B cells, enhancing IgG production. IL-4 and IL-21 are key factors influencing memory B cell formation, with IL-21, produced by T follicular helper cells, specifically promoting germinal center memory. Meanwhile, IL-15 and IL-7 play pivotal roles in memory T cell development, with IL-7 making a lesser but notable contribution to central memory T cell formation. Although these cytokines play major roles in memory formation, they are not the only factors involved in this complex process. IL-7 is produced by fibroblastic reticular cells, thymic epithelial cells, bone marrow stromal cells, and lymphatic endothelial cells within both the primary and secondary lymphoid organs. IL-15 is produced by APCs, with its production significantly upregulated in response to IFN-I [83]. Building on the foundation of systems immunology and multi-omics analysis, recent advances in synthetic biology and nanotechnology are reshaping vaccine design and delivery. Nanoparticles such as LNPs, VLPs, poly (lactide-co-glycolide) (PLGA), and caged protein nanoparticles facilitate MHC class antigen presentation by mimicking antigen size and spatial conformation. This structural mimicry, coupled with their ability to target specific dendritic cell (DC) receptors, optimizes antigen uptake, processing, and subsequent T cell activation [35].

Functionalizing NP surfaces with specific ligands enables targeted delivery to distinct APC subsets. For example, coupling NPs with anti-CD169 antibodies facilitates macrophage targeting, while CLEC9A antibodies direct NPs to conventional type I DCs. Coupling CLEC9A antibodies with NPs targets conventional type I DCs. Additionally, NPs sized between 50 and 100 nm preferentially accumulate in follicular dendritic cells, promoting durable, high-affinity humoral responses—a process further enhanced by increasing NP antigenic valence and glycosylation. Multivalent NP designs can also engage and activate B cells directly, supporting robust antibody production. Moreover, NPs can be engineered to facilitate cross-presentation, such as through membrane fusion mechanisms that enable antigen escape from endosomes, enhancing CD8+ T cell responses [35]. Integrating machine learning (ML) predictive models with multi-omics data enhances our understanding of the role of NPs in antigen presentation and APC targeting, supporting vaccine optimization before in vivo trials. In a 2023 study, Shuaib and colleagues analyzed nasopharyngeal swab samples from COVID-19 patients, dividing them into two cohorts based on the presence or absence of the R203K and G204R (KR) mutations in the SARS-CoV-2 nucleocapsid, with healthy individuals as controls [84].

The goal was to determine whether these mutations drive a stronger inflammatory response. The impact of the KR mutation on immune responses was further examined through transcriptomic and proteomic analyses of cells incubated with VLPs expressing KR nucleocapsids. Results showed that KR nucleocapsids alone triggered a significantly higher expression of immune and inflammatory response genes, including cytokines and interferon-stimulated genes (ISGs), and upregulated immune response proteins like ISG15/20, IFIT1/2/3/5/M3, IFI16/44, OAS3, and MX1 from the ISG family, alongside STAT1/2, IRF6, and IRF7 from the interferon-regulated group [84]. Additionally, KR mutations induced elevated cytokine levels (IFN-γ, IL-8, IL-6, and IL-1) and increased the neutrophil-to-lymphocyte ratio—a marker linked to COVID-19 severity [84,85]. These findings provide insight into COVID-19 pathogenesis and could inform future vaccine design. In 2022, Wang and colleagues accelerated mRNA vaccine optimization using an ML-driven approach for lipid LNP delivery systems. They analyzed 325 mRNA vaccine LNP formulations alongside the corresponding IgG titers. Remarkably, the algorithm identified key ionizable lipid substructures. Animal experiments confirmed that LNPs incorporating DLin-MC3-DMA (MC3) as the ionizable lipid induced higher efficiency in mice, aligning with the model predictions. This marked the first experimentally validated predictive model, later refined through integration with molecular modeling [86,87,88].

In 2024, Maharjan and colleagues conducted a study focusing on optimizing microfluidic conditions and lipid mix ratios for messenger RNA-lipid nanoparticles (mRNA-LNPs), although immunogenicity was not assessed. They evaluated key parameters, including particle size (PS), polydispersity index (PDI), zeta potential, pKa, heat trend cycle, encapsulation efficiency (EE), recovery ratio, and encapsulated mRNA. The results demonstrated that a self-validated ensemble model outperformed XGBoost and Bayesian optimization, achieving prediction accuracies of over 97% and 94%, respectively, while also aligning more closely with the actual experimental data [89]. In a study in 2024 by Suyash and colleagues on designing a multi-epitope vaccine for Marburg virus (MARV), they first identified immunogenic proteins from seven key MARV proteins that were capable of triggering B and T cell responses. Computational analysis confirmed the non-allergenic nature and strong antigenicity of the selected proteins. VLPs were then optimized using machine learning, although no experimental validation was performed. The in silico results provided promising leads for future in vitro and in vivo studies [90].

Similarly, in 2024, Prosper and colleagues conducted an in silico study focusing on optimizing VLP vaccine protein components (chimVLP) for SARS-CoV-2. Molecular dynamics analysis (proteomics) compared mutated and wild-type VLP proteins. Their findings indicated that while the mutant virus proteins showed slight allergenicity (except for VP2), they remained non-toxic, with an antigenicity predictive value of 0.55, according to the VaxiJen v.2.0 server [91]. An in silico study conducted in 2024 by Garmeh Motlagh and colleagues evaluated the receptor binding domain (RBD) of a SARS-CoV-2 monomeric spike protein combined with a ferritin-based nanoparticle, using molecular docking, molecular dynamics simulations, and immune simulations [92]. The study employed the root mean squared deviation (RMSD), root mean squared fluctuations (RMSF), principal component analysis (PCA), the cross-correlation matrices of the Cα atoms, solvent-accessible surface area (SASA), and the C-IMMSIM server to comprehensively assess structural stability, flexibility, and immunogenicity. The results demonstrated the robust stability and structural integrity of the nanoparticle complex, alongside a notable rise in B cell, CD4+, and CD8+ T cell populations following each dose. Moreover, elevated levels of IgG, IgM, IFN-γ, and IL-2 were observed after each immunization. Despite these promising immunogenic responses, the durability of the immune response remained limited when tested in a three-dose regimen with 30-day intervals over a two-month period. This suggests that further optimization—including structural refinement and in vivo experimentation—is essential to validate and enhance the platform’s performance [92].

As mentioned earlier, Mayer and colleagues conducted a study in 2022, developing the first mRNA vaccine targeting an intracellular bacterium, *Listeria monocytogenes*. They employed proteomics, specifically immunopeptidomics, to identify the surface antigens presenting on infected cells, guiding the design of an mRNA vaccine delivered via a lipid nanoparticle platform. The results highlighted LMON_0149 as a promising vaccine target. Mice that were vaccinated with this formulation demonstrated detectable antigen-specific IFN-γ responses—a key indicator of CD8+ T cell activation—alongside significant protection in both the liver and spleen [61]. These computational approaches pave the way for further model development, potentially correlating with the immunogenicity of pathogens such as influenza, CMV, *Plasmodium falciparum*, and *Chlamydia trachomatis* (Table 4).

The reviewed studies demonstrate a consistent shift toward using computational tools to enhance predictive and personalized vaccine design. Although the methodologies differ, ranging from transcriptomics to machine learning-guided lipid nanoparticle optimization and in silico epitope mapping, they share a unified aim to streamline and improve antigen selection, delivery mechanisms, and immune profiling. These advancements cover the way for rationally designed platforms that can be customized for specific pathogens, immune environments, and even individual patient genotypes, signaling a transformative move from traditional experimental approaches to systems-driven vaccine engineering.

## 6. Synergistic Integration of the Three Pillars

Based on different research studies, synthetic nanoparticles in immunotherapy and vaccinations will greatly affect public health. Antibiotic resistance is one of the main global issues nowadays, which has promoted new diagnostic techniques, as well as alternative therapeutic and preventative strategies. The successful treatment of infectious diseases depends on early detection, and recently, developed applications based on nanotechnology have provided a more sensitive and effective diagnostic model [93]. In the past three years, nucleoside-modified mRNA-lipid nanoparticle (mRNA-LNP) vaccines have been developed against the SARS-CoV-2 Wuhan-Hu-1 strain to stimulate humoral and cellular immunity. Although rare, there are reports of adverse effects from COVID-19 mRNA vaccines, such as acute myocardial infarction, Bell’s palsy, cerebral venous sinus thrombosis, stroke, and thrombosis with thrombocytopenia syndrome. Codon optimization plays a crucial role in the antigen design of mRNA therapeutics and vaccines through multiple aspects of the target protein expression: mRNA abundance, mRNA stability, translational efficiency, and correct protein folding [94].

Synonymous codons and their cognate tRNAs occur at different frequencies across proteins, their domains, and the organisms from which they originate [95,96]. A previous study by Chih-Jen Lai et al. showed that spike protein codon optimization using *Herpes Simplex Virus-1* (HSV) codons, instead of human codons, increased immunity. This optimization resulted in higher levels of spike-specific antibodies, neutralizing antibodies (NAbs), and better protective immunity against lethal SARS-CoV-2 infection. Additionally, the HSV codon-optimized mRNA vaccines for the Delta and Omicron variants showed improved efficacy compared to human codon-optimized vaccines. The results suggest that using viral codon optimization platforms could lead to the development of mRNA therapeutics with lower dosages and fewer side effects [97]. A study by Bapurao Surnar et al. showed that IVM (ivermectin) can accumulate in the circulation at a greater concentration when administered via synthetic NPs, and the initial findings indicated that NP-delivered IVM can specifically target the Zika virus (ZIKV) nonstructural 1 protein. The long-term storable formulation of IVM-nanoparticles in a dry powder state for inclusion in capsule form and as a cryoprotectant, including frozen forms, showed encouraging results with possible clinical significance. Additionally, the initial in vitro research showed that ivermectin crosses the placental barrier, making it dangerous for pregnant ZIKV patients. In contrast, the ivermectin-loaded nanoparticle showed no discernible placental barrier-crossing behavior, suggesting that it might be appropriate for this population [98].

Based on a case study by Peter G. Kremsner et al., conducted on 245 adults aged 18 to 60 years old who were divided into vaccine injection and placebo injection groups, the research showed that two doses of the CVnCoV vaccine had acceptable reactogenicity and were safe. Immune responses at levels similar to those seen in recovered antibodies from COVID-19 patients were induced by a dosage of 12 μg [99]. In recent years, nanotechnology has developed quickly in the field of healthcare research, and nanovaccines hold great promise for resolving the issues seen with traditional peptide-based vaccines, as follows. First, APC phagocytosis and lymph node (LN) retention can affect nanoparticles. Second, the administration of antigens and adjuvants by structured nanovaccines promotes strong antigen presentation activation. Third, the pharmacokinetic characteristics of encapsulated antigens and adjuvants, such as prolonging drug circulation time and delaying drug breakdown, may be markedly enhanced by nanotechnology [96]. With basic chemical synthesis techniques, a study created a novel self-assembly vehicle-free multi-component antitumor nano-vaccine (SVMAV) that stimulated strong antitumor immune responses with fewer harmful side effects. The SVMAV is made up of antigen peptides, R848, and static. Following subcutaneous injection, the SVMAV homed in on LNs and was caught by the LN-residing DCs, which, in turn, stimulated the CD8+ T cells, facilitated DC maturation and antigen cross-presentation, and ultimately began the targeted destruction of tumor cells. Additionally, this method could be used to administer customized cancer vaccinations. Considering all these factors, our research presents a compelling and feasible strategy for enhancing the therapeutic efficacy of vaccine-based cancer immunotherapy through the application of nanotechnology. A self-assembly nanovaccine, containing the TLR7/8 agonist and STAT3 inhibitor, enhances tumor immunotherapy by augmenting the tumor-specific immune response. Tumor cells, however, have detrimental impacts on immune activities in general, including the release of metabolic products, immunosuppressive cytokines, and ligands for immunological checkpoint receptors. Furthermore, neo-antigen-specific cytotoxic T lymphocytes (CTLs) are inherently uncommon in a variety of cancer types. Neoantigen-specific CTLs, for instance, make up less than 0.001% of the peripheral T cell population in individuals with colorectal cancer [100,101].

The vaccine-induced activation of tumor-specific immune responses is a highly desirable approach to address these issues. Personalized tumor neoantigens can now be quickly identified, thanks to recent developments in next-generation sequencing (NGS) and analytics, especially for tumors with modest mutation burdens or with no shared neoantigens [102]. For the time being, there are two widely used COVID-19 vaccines in the world, which are mRNA-based and encapsulated in lipid nanoparticles: mRNA-12734 [103] and BNT162b25 [104], from Moderna and Pfizer-BioNTech’s lipid-based nanoparticle vaccine. Compared to conventional vaccines, these showed high efficiency (above 94%), which established their combat potential for future SARS-CoV-2 variants [105,106]. In contrast, there are nanoparticle-based DNA vaccines, which, compared to mRNA vaccines, are more cost-effective, easy to generate, and perform with greater stability. Human anti-tumor immunity efficiency is related to T cells, which attack cancer-specific neoantigens. Tumor cells produce these specific protein markers, which do not exist in normal tissues. Therefore, they trigger a strong immune response through the avoidance of natural body tolerance mechanisms [107,108]. According to a study by Patrick A. Ott et al., even in the absence of previous or current treatments, a personalized neoantigen vaccination is effective, safe, and capable of eliciting robust T cell responses in cancer patients. This can move beyond the two main challenges in cancer treatment: tumor incompatibility and targeting tumor tissue versus healthy tissue. It has been demonstrated that the vaccine causes the production of new T cell clones that are able to identify processed antigens along with the patient’s own tumor cells, as well as several patient-specific neoantigens. This indicates that a range of malignant clones may be targeted by the vaccination, addressing tumor diversity and reducing the possibility of tumor escape as a result of antigen loss [109].

**Figure 3:**
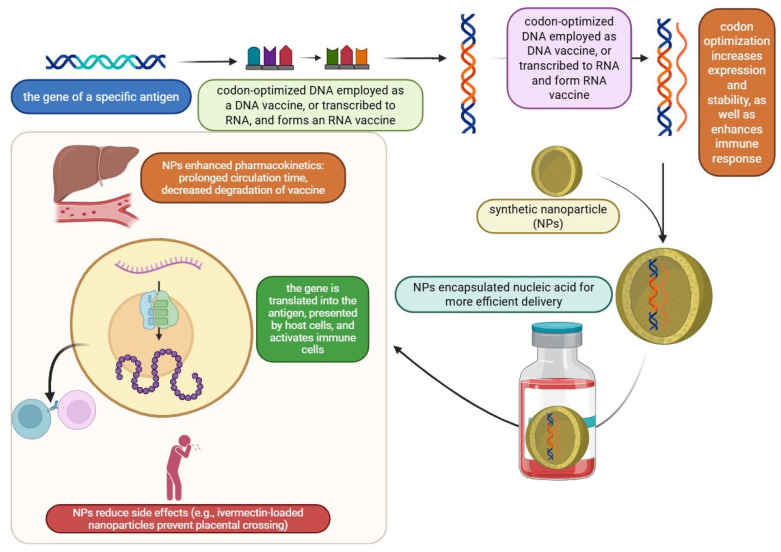
Three-pillar strategy for next-generation vaccines: nanoparticles, codon optimization, and mRNA/DNA technologies.

The development of vaccines is being entirely altered by the use of artificial intelligence (AI), which is bringing about notable breakthroughs at every level from discovery to dissemination. Through antigen identification, immune response prediction, and vaccine design optimization, AI speeds up the vaccine discovery process. By facilitating real-time data monitoring and enhancing participant enrollment, AI also improves clinical trials. AI enhances process efficiency, equipment maintenance, and quality control in vaccine manufacturing. It also streamlines logistics and cold-chain management for distribution, guaranteeing a quicker and more equitable vaccine supply. Notwithstanding these developments, issues like data integration, model transparency, and moral and legal dilemmas still exist. Personalized vaccines and enhancing global health equity will be the main topics of future research. Working together, AI specialists, vaccine creators, and medical institutions can improve global health outcomes and expedite illness response [110]. A comprehensive review of next-generation vaccine innovations is shown in Figure 3 and Table 5.

**Table 5:** Selected clinical trials and approved vaccines based on next-generation platforms.

Platform Type	Product/Trial Name	Technology Used	Target Disease	Clinical Phase/Status
mRNA-based (LNP)	BNT162b2 (Pfizer–BioNTech)	Synthetic mRNA + lipid nanoparticles	COVID-19	Approved (EMA, FDA)
saRNA	ARCT-154 (Arcturus)	Self-amplifying RNA + LNP	COVID-19	Phase 3
DNA Vaccine	INO-4800	Synthetic plasmid DNA	COVID-19	Phase 3
Nanoparticle Protein-based	NVX-CoV2373 (Novavax)	Recombinant protein + Matrix-M (saponin NP)	COVID-19	Approved (e.g., WHO, EU)
VLP-based	Mosquirix (RTS,S/AS01)	Hepatitis B-based VLP + AS01 adjuvant	Malaria	Approved (WHO, 2021)
Personalized Neoantigen	mRNA-4157/V940 + Keytruda	mRNA encoding patient-specific tumor neoantigens	Melanoma (Cancer)	Phase 2 (Positive results)

LNP: Lipid nanoparticle, VLP: virus-like particle, saRNA: self-amplifying RNA, AS01: MPL + QS-21 liposomal adjuvant (Table 6 and Table 7).

## 7. Integrated Conceptual Framework: Synergistic Interaction of Synthetic Biology, Nanotechnology, and Systems Immunology

Synthetic biology, nanotechnology, and systems immunology each bring distinctive strengths to the development of next-generation vaccines. When integrated, they create a synergistic framework that amplifies immunogenicity through interconnected molecular and cellular processes. On the molecular front, synthetic biology facilitates the programmable design of antigens, whether mRNA, DNA, or peptides, utilizing advancements like codon optimization, epitope mapping, and self-amplifying RNA systems. These engineered antigens are delivered using nanocarriers such as LNPs, VLPs, or polymer-based systems. These carriers stabilize the antigens, protect them from degradation, and ensure efficient delivery to APCs. Many nanocarriers also function as adjuvants by supporting depot formation, promoting membrane fusion, or directly activating PRRs. At the cellular level, this delivery system ensures efficient antigen processing and presentation through both MHC class I and II pathways, strengthening the CD4+ and CD8+ T cell responses. Systems immunology provides an additional layer by decoding the host immune response through multi-omics approaches (transcriptomics, proteomics, and epigenetics). This enables iterative vaccine optimization by identifying the ideal antigens, modifying immune responses (e.g., Th1 vs. Th2), and personalizing regimens according to individual immune profiles. Together, these disciplines form a dynamic feedback loop: synthetic biology can design optimized antigens, nanotechnology facilitates precise delivery, and systems immunology analyzes the immune outcomes to guide further modifications. This integrated approach supports precision immunoprophylaxis, resulting in vaccines that are safer, more effective, and adaptable to emerging pathogens or unique immune landscapes [114,115] (Table 8).

## 8. Challenges and Ethical Considerations

The broad field of nanomedicine makes use of nanotechnology for therapeutic purposes. It entails the creation, advancement, and use of nanoscale tools and materials for illness detection, management, and prevention. Additionally, it covers a broad spectrum of uses, such as medicinal substances, diagnostic instruments, and drug delivery systems [116]. The safety assessment of nanomaterials and nanodevices is one of the main regulatory concerns in nanomedicine. Because of their small size, nanomaterials have unique physicochemical properties that can lead to different biological interactions from those with conventional materials. Regulatory agencies, including the European Medicines Agency (EMA) and the U.S. Food and Drug Administration (FDA), have been working to establish guidelines for evaluating the safety of and conducting risk assessments of nanomedicine products. These guidelines center on the characterization, toxicity, and environmental impact of nanomaterials, as well as the requirement for appropriate preclinical and clinical studies to evaluate their safety and efficacy [117].

Public health plays a critical role in the implementation of nanotechnology, particularly in the context of medicine. Nanomedicine, due to its direct application in healthcare, faces heightened public scrutiny. Avoiding negative public perception influences the acceptance of nanomedicine significantly [118]. People’s fear of death and their concern over medical conditions result in less difficulty regarding the acceptance of novelty in this area. However, any accident related to nanomedicine may cause widespread distrust in the field [119].

A major regulatory challenge in nanomaterials is the lack of standardized characterization techniques, due to their diverse properties. Developing and adopting standardized protocols for assessing key attributes like physicochemical properties, their stability, and potential aggregation or transformation is essential. These methods will improve the accuracy of any safety and efficacy evaluations and streamline regulatory approval for nanomedicine products [120]. The absence of proper classification and regulatory processes specific to these products is another significant regulatory hurdle in nanomedicine. The special qualities and complexity of nanomedicines are frequently not adequately addressed by conventional frameworks. Regulatory bodies are creating certain guidelines in response. The FDA, for instance, has released guideline documents that list crucial factors to take into account when assessing the efficacy and safety of applications that include nanomedicine [121]. Although VLP-based vaccines have the potential to protect against diseases such as the Zika virus, HCV, HBV, and HPV, their commercialization is hindered by a number of issues. Researchers are enhancing formulations to navigate these obstacles by refining adjuvants, stabilizers, administration routes, and delivery systems (such as liposomes and microparticles) in order to lower dosage and prolong shelf life without the need for cold chains. Additionally, efforts are being made to develop more reasonably priced vaccinations for LMICs, or low- and middle-income nations. However, prior experiences, like the delayed availability of HPV vaccines because of their high initial costs, make financial barriers a crucial problem that needs to be resolved in order to guarantee fair distribution around the world [122].

### 8.1. Regulatory Challenges

The intersection of synthetic biology, nanotechnology, and systems immunology has highlighted regulatory uncertainty, as existing frameworks were established primarily with traditional vaccines in mind. Agencies such as the FDA and EMA lack standardized pathways that are tailored to evaluating highly modular or self-amplifying constructs. For instance, the development of mRNA vaccines necessitated emergency use authorizations (EUAs), given the absence of prior precedent; their approval relied heavily on accelerated clinical trials, bridging data from similar platforms, and real-time audits of the production process. In addition, platforms integrating novel delivery methods such as LNPs or VLPs often encounter delays, due to the extensive testing required for toxicity, biodistribution, and adjuvant compatibility. Regulatory approaches must evolve to address the unique challenges posed by these technologies. Core questions include how synthetic components interact with host immune systems, the potential long-term risks associated with engineered vectors, and the implications of in silico-driven vaccine personalization on population-wide efficacy and equity [123].

### 8.2. Manufacturing Scalability and Complexity

Manufacturing next-generation vaccine platforms presents significant hurdles, due to intricate supply chains and scaling difficulties. Self-amplifying RNA (saRNA) vaccines, for example, demand precise enzymatic transcription and purification steps, while VLPs rely on expression in advanced systems like insect or mammalian cells, requiring stringent glycosylation control. Nanoparticle formulation, particularly for cold chain-reliant LNPs, adds another layer of complexity to production logistics. Globally scaling these technologies remains challenging because of the limited availability of standardized GMP-compatible microfluidic production systems and the high cost of essential materials such as ionizable lipids and synthetic polymers. Unlike conventional recombinant protein vaccines, these platforms also necessitate multi-step quality control processes to ensure consistency in batch production, antigen stability, and immune response performance [124].

### 8.3. Cost Implications and Global Access

Despite their remarkable potential, modular vaccine platforms come with substantial up-front research, development, and infrastructure costs. The cost-per-dose of mRNA- and nano-enabled vaccines is significantly higher than that of traditional inactivated or protein subunit vaccines, limiting their widespread adoption in low- and middle-income countries (LMICs). Furthermore, personalized or stratified vaccine approaches that are informed by systems immunology may not be feasible on a global scale without affordable and scalable diagnostic systems [125].

### 8.4. Case Studies on Regulatory Approval

The COVID-19 pandemic offered valuable insights into regulatory processes. The authorization of Pfizer-BioNTech’s and Moderna’s mRNA vaccines required unprecedented regulatory coordination through rolling reviews and post-marketing measures to monitor rare adverse events. Similarly, the RTS,S/AS01 malaria vaccine—the first to utilize the AS01 adjuvant system (a combination of liposomes, MPL, and QS-21)—required over a decade of data collection to satisfy the safety and efficacy benchmarks, primarily due to its novel adjuvant component. These case studies underscore the critical importance of engaging with regulators early in the development process, maintaining platform master files for efficient evaluation, and establishing robust pharmacovigilance systems. Moving forward, vaccine developers must strike a balance between innovation and adherence to the existing regulatory frameworks to enable timely, equitable access to these advanced technologies [126].

## 9. Conclusions

The landscape of vaccinology is being reshaped by the integration of synthetic biology, nanotechnology, and systems immunology. In contrast to traditional methods, next-generation platforms enable precise design, efficient delivery, and personalized immune engagement. mRNA and DNA vaccines, which have been improved through codon optimization and lipid nanoparticle delivery, have shown rapid development and high immunogenicity, as evidenced during the COVID-19 pandemic. Nanoparticle-based systems not only protect antigens but actively enhance antigen presentation via the MHC I and II pathways, leading to better T and B cell responses. Additionally, systems immunology uses multi-omics data and AI-driven modeling to unravel the intricacies of immune reactions, aiding in the rational design of vaccines. These technologies collectively offer solutions to challenges such as immunogenicity, delivery challenges, and antigen instability. Despite problems such as regulatory standards and equitable access, the potential of these platforms is clear. They indicate a significant change toward vaccines that are quicker to develop, safer, adaptable, and more efficient for combating infectious diseases and cancer.

## Figures and Tables

**Table 1:** Clinically approved adjuvants and their mechanisms of action.

Adjuvant	Composition/Type	Mechanism of Action	Approved Vaccines
Aluminum salts (Alum)	Mineral salts (e.g., aluminum hydroxide)	Creates a depot effect and activates inflammasomes (e.g., NLRP3), enhancing APC uptake and Th2 responses	Hepatitis A/B, DTaP, HPV
MF59	Squalene-based oil-in-water emulsion	Enhances antigen uptake and recruitment of APCs; promotes cytokine release (IL-6, MCP-1)	Influenza (e.g., Fluad)
AS03	α-tocopherol + squalene emulsion	Stimulates cytokine production (e.g., IL-6) and promotes antigen presentation	H1N1 (e.g., Pandemrix)
AS01	Liposome-based with MPL + QS-21	Activates TLR4 and promotes Th1-biased immunity through APC maturation	Shingrix (herpes zoster), Malaria (RTS,S)
CpG 1018	Synthetic TLR9 agonist (DNA oligo)	Directly activates plasmacytoid dendritic cells and B cells, promoting Th1 responses	HEPLISAV-B (hepatitis B)
Poly I:C	Synthetic dsRNA (TLR3 agonist)	Mimics viral RNA, stimulating IFN production and cytotoxic T cell responses	(Experimental; not yet widely approved)

**Table 2:** Synthetic biology-based vaccine platforms and their mechanisms.

Platform	Mechanism	Examples	Advantages	Limitations	Ref.
mRNA vaccines	Encodes antigens via synthetic RNA expressed in host cells	Pfizer-BioNTech, Moderna	Rapid production, customizable, strong immune response	Requires cold chain, limited stability	[22,23,24,25,26,43,44]
DNA vaccines	Synthetic plasmid DNA encoding antigens delivered into host cells	INO-4800	High stability, easy storage	Lower efficacy in humans, requires special devices	[26,27,28,42,45]
Mineral salts	↑ the immunogenicity of some vaccines, such as pertussis, diphtheria, poliomyelitis	aluminum adjuvants	↑ Immune response, improved antigen delivery, and development of new vaccines	Potential side effects, limited human use, and restricted approval	[34,38,46]
Emulsions	↑ the immunogenicity of some vaccines such as influenza.	MF59 AS03	↑ Immune response, improved antigen delivery, and the development of new vaccines.	Potential side effects, limited human use, and restricted approval	[34,36,37]
Codon optimization	Process of modifying the codons in a gene to enhance protein expression in a specific organism	CodaVax-H1N1 CodaVax-RSV CDX-005	↑ Protein production, no in-depth viral function knowledge needed, faster response to a reduced risk of viral reversion	Codon usage bias variability, time-dependent process	[29,30,31,32,33,47]
Self-amplifying RNA vaccines	Synthetic RNA replicates inside cells producing more antigens	ARCT-154	Lower dose needed, and stronger immune response	Novel platform, with safety still under evaluation	[41,42,48]

↑: Increase.

**Table 3:** Nanomaterials used in vaccine platforms: their properties and applications.

Studies (Year)	Nano Material	Properties	Application	Advantages	Challenges	Ref.
Sun (2023)	Protein-based polymeric nanoparticle	Glycoprotein	EBV	Durable humoral immunity (Nab, IgG, IgA)	No cellular immunity—autoimmunity	[54]
Widge (2023)	Protein-based nanoparticle	Hemagglutinin head	Influenza	Durable humoral immunity (Nab, IgG)—cross-reactivity among group 1 influenza	Limited response against group 2 influenza	[56]
Ramirez (2023)	Protein-based nanoparticle	Outer membrane protein antigen	*Coxiella burnetii*	Strong cellular and humoral immune response	Not evaluated in humans—low durability of responses	[57]
Martínez-Pérez (2021)	Lipid-protein nanoparticles	Protein antigen	*Mycobacterium tuberculosis*	Memory T cell induction	Low durability of responses	[59]
Essink (2024)	Lipid-based nanoparticle	mRNA	SARS-CoV-2	Cross-reactivity	Small sample size	[60]
Mayer (2022)	Lipid-based nanoparticle	mRNA	*Listeria monocytogenes*	Strong cellular response	Not evaluated in humans	[61]
Wan (2024)	Lipid-based nanoparticle	circRNA	Influenza virus and SARS-CoV-2	Robust cross reactivity	Not evaluated in humans—allergic-type reaction	[62]
Friedman Klabanoff (2024)	Liposomes	full-length recombinant Circumsporo-zoite	*Plasmodium falciparum*	Favorable safety and tolerability—high avidity of full-length and C-terminal region antibodies	Non-protective immunity	[65]
Pollock (2024)	Liposomes	Outer membrane molecule	*Chlamydia trachomatis*	Humoral (IgG and IgA) and cellular immune response	Small sample size	[66]
Gobeil (2021–22)	Virus-like particle	Full-length Spike protein	SARS-CoV-2	Robust, durable, and cross-reactive humoral immunity—durable Th1 and Th2 response induction	Allergic-type reaction	[68,69]
He (2024)	Virus-like particle	Pan-epitope peptide TBT	*Flavivirus*	High uptake rate	Not evaluated in humans	[70]
Langley (2024)	Enveloped virus-like particle	Glycoprotein	CMV	Sustained humoral responses	Low vaccine dose	[71]
NAb = neutralizing antibody						

**Table 4:** Systems immunology tools and their contributions to vaccine design.

Studies (Year)	Tool	Description	Contribution	Challenges	Examples	Ref.
Shuaib (2023)	Transcriptomics and Proteomics in Mutation Analysis	Gene expression profiling and protein-level immune analysis of viral mutations	Reveals how KR mutations in SARS-CoV-2 nucleocapsid drive a stronger inflammatory response	Requires further exploration to link findings to vaccine development	SARS-CoV2	[84]
Wang (2022)	ML-Driven LNP Optimization	Predictive modeling of LNP formulations for mRNA delivery	Identifies optimal LNP structures for enhanced vaccine efficiency	Integration with molecular modeling required further refinement	-	[86]
Maharjan (2024)	Microfluidics and Ensemble Models	Fine-tuning microfluidic conditions and lipid mix ratios for mRNA-LNP formulation	Achieves high prediction accuracy in particle properties	Immunogenicity not assessed	-	[89]
Suyash (2024)	Computational Epitope Design	Identification of immunogenic proteins for vaccine development	Enables rational design of multi-epitope vaccines	No in vitro/in vivo experimental validation performed	Marburg virus	[90]
Prosper (2024)	Molecular Dynamics in VLP Design	Structural analysis of VLP proteins	Assesses the antigenicity and allergenicity	Predictive models require in vitro/in vivo validation	SARS-CoV2	[91]
Garmeh Motlagh (2024)	RBD-Nanoparticle Analysis	Structural and immunogenicity assessment of SARS-CoV-2 RBD-ferritin nanoparticles	Evaluates the stability, flexibility, and immune response	Limited immune response durability; requires further optimization and in vivo validation	SARS-CoV2	[92]
Mayer (2022)	Immunopeptidomics for mRNA Vaccine Targeting Bacteria	Peptide profiling of infected cells to guide vaccine design	Identifies the antigenic targets for intracellular bacterial vaccines	Translating findings to human applications remains a challenge	*Listeria monocytogenes*	[61]

**Table 6:** Synergistic integration of synthetic biology, nanotechnology, and systems immunology in next-generation vaccines.

Vaccine Platform	Synthetic Biology Contribution	Nanotechnology Contribution	Systems Immunology Contribution	Example	Outcome	Ref.
mRNA-LNP Vaccines	HSV-1 gB codon_optimized mRNA sequences for SARS-CoV-2 spike protein expression	Encapsulation of optimized mRNA in lipid nanoparticles to protect mRNA, enhance delivery, ensure endosomal escape, and increase intracellular expression of the antigen.	Induction of strong humoral and cellular immune responses, including high titers of neutralizing antibodies and robust T cell activation against SARS-CoV-2 and its variants.	HSVgB codon-optimized Delta Spike mRNA vaccine co-expressed with HSV-1 ICP27.	Spike-specific IgG, neutralizing antibodies were markedly increasing, with greater immunological response at reduced dosages of mRNA. Decreased likelihood of adverse effects.	[97]
Self-Assembling Nanovaccines	Combination of TLR7/8 Agonists and STAT3 Inhibitors	Self-assembling vehicle-free multicomponent antitumor nanovaccine (SVMAV)	A multi-layered approach to activate and balance various parts of the immune system to improve the immune response against tumors.	HPV vaccine (Gardasil), experimental influenza vaccines	Enhanced tumor control, a stronger immune response against malignancies, and the possibility of better results from cancer immunotherapy.	[111]
DNA-Nanoparticle Vaccines	recombinant HexaPro spike plasmid DNA against SARS-CoV-2 Gamma lineage	Nanoparticles to boost cellular delivery and uptake	Modeling immune response to improve efficiency of dosing and delivery	DNA nanoparticle vaccine against SARS- CoV-2 ZyCoV-D	Ameliorate transfection efficiency and stronger immune activation	[112]
Synthetic Immune Circuits	Logic-based receptor design (e.g., SynNotch), express gene control	Nanocarriers for targeted transfer of genetic circuits	Modeling and analysis of immune responses to optimize design	SynNotch T cells activated only in the presence of the tumor antigen	Increased specificity, decreased side effects, and better control of immune functions	[113]
Personalized Cancer Vaccines	Neoantigen identification and mRNA synthesis	Lipid nanoparticles for personalized delivery	Patient-specific immune profiling to tailor vaccines	BioNTech mRNA-based personalized cancer vaccines	Individualized immune responses, improved tumor targeting	[109]

**Table 7:** Comparative analysis of the various vaccine platform strategies.

Platform Type	Immunogenicity (H/C) *	Manufacturing Complexity	Stability	Cost	Notable Advantages	Key Limitations
mRNA (LNP-based)	High/Moderate	Moderate to High	Low (unless lyophilized)	High	Rapid development, strong cellular response	Cold chain needed; limited long-term data
DNA vaccine	Moderate/Low	Low	High	Low	Stable, easy to store	Weaker human efficacy; delivery device required
Peptide (adjuvanted)	Low/Moderate	Low	High	Low–Moderate	Safe, defined antigens	Needs adjuvant, often weak CD8+ T-cell activation
Protein subunit (e.g., recombinant)	Moderate/High	Moderate	Moderate	Moderate	Proven platforms (e.g., HBV, HPV)	Requires adjuvants; slower production
VLP (Virus-like particle)	High/High	High	Moderate	High	Multivalent display; strong B and T cell responses	Complex manufacturing; scalability challenges
saRNA (Self-amplifying)	High/High	High	Low	Moderate–High	Lower dose needed; enhanced expression	Still under evaluation; safety and scale-up ongoing
Nano-based (liposome, polymer)	High/Variable	High	Moderate	Moderate–High	Tunable targeting; co-delivery of antigen + adjuvant	Complex formulation; regulatory challenges
Synthetic biology + NP + systems immunology	Very High/High	Very High	Moderate	High	Precision-targeted, personalized, and adaptable platforms	Requires infrastructure and advanced data analytics

* H = humoral immunity; C = cellular immunity.

**Table 8:** Case studies of next-generation vaccine platforms across diverse pathogen classes and disease contexts.

Pathogen/Disease Type	Target	Vaccine Platform	Key Outcome
Viral	SARS-CoV-2 (Omicron)	mRNA in lipid nanoparticles (CV0501)	Strong neutralizing antibody and Th1-skewed T cell responses
Bacterial	*Listeria monocytogenes* (LMON_0149 antigen)	mRNA in lipid nanoparticles	Robust CD8⁺ T cell activation and antigen-specific IFN-γ production
Parasitic	*Plasmodium falciparum* (Circumsporozoite protein)	Protein subunit + GLA-LSQ nanoliposome	Elevated IgG titers with a favorable safety profile
Cancer (Non-Infectious)	Tumor neoantigens	Personalized mRNA vaccine + LNP	Induction of tumor-specific T cell responses and tumor regression
Autoimmune Conditions	Experimental autoimmune encephalomyelitis (EAE)	Tolerogenic nanoparticle-based vaccine	Reduction in disease severity via antigen-specific tolerance

## Data Availability

The corresponding author will provide the datasets created during and/or analyzed during the current investigation upon reasonable request.

## Glossary of Key Technical Terms

Term	Definition
Codon Optimization	The modification of gene codons to improve the efficiency of protein expression in the target host organism.
Virus-like Particles (VLPs)	VLPs are structures that resemble viruses but lack genetic material, making them a safe and effective tool for triggering immune responses to vaccines.
Self-amplifying RNA (saRNA)	A synthetic RNA platform that replicates within host cells, producing higher antigen levels from lower vaccine doses.
Pattern Recognition Receptors (PRRs)	Cellular receptors that recognize pathogen-associated molecular patterns (PAMPs) to trigger immune responses.
Multi-omics	An integrative approach combining datasets from various “omics” fields, such as genomics, transcriptomics, and proteomics.
Codon Usage Bias	The preferential usage of certain synonymous codons over others in a gene, which can affect protein translation efficiency.
Nanovaccine	A vaccine platform that utilizes nanoparticles to deliver antigens directly and efficiently to immune cells.
Adjuvant	A substance added to vaccines to enhance the body’s immune response to the presented antigen.
Transcriptomics	The comprehensive analysis of all RNA transcripts in a cell or tissue used to study gene activity and regulation.
Immunopeptidomics	A proteomics subfield that is focused on identifying the peptides presented by MHC molecules to inform precise vaccine design.
